# Differential Susceptibility of Catheter Biomaterials to Biofilm-Associated Infections and Their Remedy by Drug-Encapsulated Eudragit RL100 Nanoparticles

**DOI:** 10.3390/ijms20205110

**Published:** 2019-10-15

**Authors:** Vivek Kumar Pandey, Kumar Rohit Srivastava, Gufran Ajmal, Vijay Kumar Thakur, Vijai Kumar Gupta, Siddh Nath Upadhyay, Pradeep Kumar Mishra

**Affiliations:** 1 Department of Chemical Engineering & Technology, Indian Institute of Technology (Banaras Hindu University) Varanasi, Varanasi, Uttar Pradesh 221005, India; rohit.rs.che14@itbhu.ac.in (K.R.S.); snupadhyay.che@itbhu.ac.in (S.N.U.); 2 Department of Pharmaceutical Engineering & Technology, Indian Institute of Technology (Banaras Hindu University) Varanasi, Varanasi, Uttar Pradesh 221005, India; gufranjamal86@gmail.com; 3 Enhanced Composites and Structures Center, School of Aerospace, Transport and Manufacturing, Cranfield University, Bedfordshire MK43 0AL, UK; 4 Department of Chemistry and Biotechnology, ERA Chair of Green Chemistry, Tallinn University of Technology, 12618 Tallinn, Estonia

**Keywords:** *E. coli*, biofilm, nanoparticle-mediated drug delivery, antibiotics, catheters, biomaterials

## Abstract

Biofilms are the cause of major bacteriological infections in patients. The complex architecture of *Escherichia coli* (*E. coli*) biofilm attached to the surface of catheters has been studied and found to depend on the biomaterial’s surface properties. The SEM micrographs and water contact angle analysis have revealed that the nature of the surface affects the growth and extent of *E. coli* biofilm formation. In vitro studies have revealed that the Gram-negative *E. coli* adherence to implanted biomaterials takes place in accordance with hydrophobicity, i.e., latex > silicone > polyurethane > stainless steel. Permanent removal of *E. coli* biofilm requires 50 to 200 times more gentamicin sulfate (G-S) than the minimum inhibitory concentration (MIC) to remove 90% of *E. coli* biofilm (MBIC_90_). Here, in vitro eradication of biofilm-associated infection on biomaterials has been done by Eudragit RL100 encapsulated gentamicin sulfate (E-G-S) nanoparticle of range 140 nm. It is 10–20 times more effective against *E. coli* biofilm-associated infections eradication than normal unentrapped G-S. Thus, Eudragit RL100 mediated drug delivery system provides a promising way to reduce the cost of treatment with a higher drug therapeutic index.

## 1. Introduction

The biomedical devices used in the management of vital health functions are the backbone of modern medicine. Catheters made of different biomaterials like polyurethane, silicone rubber, polyvinyl chloride, polypropylene, polyethylene, Teflon etc. are the most common externally implanted medical devices (EIDs) [1]. With the advancement in medical technology, a significant increase in implant-related cases of biofilm-associated infections over EIDs has emerged as a major global health challenge [2]. Proper screening for new catheter/implant materials with desirable biocompatibility and antibacterial property for prevention of biofilm formation is still a matter of research [3]. Biofilm-associated infections have become the most dangerous type of virulent factor for the majority of infectious pathogenic microorganisms. There is a high incidence of biofilm-associated infections on artificial implanted devices such as catheters, orthopedic implants, stents, contact lenses, and other electronic health support devices [4]. Chronic catheter-associated biofilm infections are frequently caused by Gram-positive as well as Gram-negative bacteria [5,6,7]. The prevention of catheter-related infections by premature removal of catheters has led to an increase in treatment cost [8].

Considerable efforts have been made to develop new antimicrobial agents for preventing biofilm-associated infections [9]. The development of new antibiotics and widespread application of existing antibiotics has led to the growth of tolerant microorganisms either by mutation of genes or by change in the mode of their proliferation [10].

Microbial communities can adhere to different surfaces using extracellular polymeric substance’s (EPS) microenvironment, composed mainly of polysaccharides, proteinaceous molecules, lipids, mucus, and some extracellular DNA, and get embedded deep inside the EPS. Such a micro-environment improves the bacterial resistance against various factors like biocides, host defense and unfavorable environmental conditions [11,12]. The microbes in the deepest layer of biofilm are resistant to antibiotics as the EPS layers limit their exposure to antibiotics. The bacteria residing in biofilm colonies are metabolically dormant, which makes them resistant to conventional biocides sometimes 1000-fold more than the planktonic forms of the same strain [6].

The antibiotic resistance may involve either production of some inhibitor enzymes or modification of the cell permeability to enhance multidrug resistance. The genes for antibiotic resistance have been reported for lateral transfer along bacterial biofilms. Failure of antibiotic treatment of biofilm-related infections makes the task of treatment extremely challenging, resulting in delayed, expensive treatment or sometimes surgical removal of the implant [13].

Nearly 80% of human bacterial infections are biofilm-associated. *E. coli, Pseudomonas aeruginosa, Staphylococcus epidermidis* and *Staphylococcus aureus* are among the most prominent causative agents. The catheter’s surface always remains in touch with biological fluids, thus providing an excellent platform for bacterial attachment and proliferation. The duration of catheterization is a major determinant for biofilm formation on all EIDs. Nearly 20% of the cases of healthcare acquired bacterial infections in acute care facilities and more than 50% in long term care facilities are catheter-associated urinary tract infections. [14]. Due to the lack of well-defined treatment protocol for biofilm-associated infections, biofilm-associated iatrogenic infections contribute significantly to patient morbidity and healthcare costs. Few possible strategies, such as targeting the regulators and eradicating biofilms, have already been investigated [15]. Bacterial cells produce and release some signaling molecules for communication to regulate a wide variety of characteristic functions including biofilm formation by the phenomenon of quorum sensing (QS) [16]. QS inhibitors have been used either for interrupting or degrading or modifying QS signals [17,18]. EPS matrix-degrading enzymes have been shown to act by the degradation of polysaccharides, DNA, or proteinaceous components in biofilm matrix [19]. Antimicrobial peptides modify the attachment, influence QS systems, and promote twitching motility [20]. Surfactants like CTAB, Tween 20, Triton X-100, rhamnolipids, etc. have also been found to be effective in lowering the surface tension to detach adherent bacterial cells leading to biofilm dispersion [21,22]. Free fatty acids tend to inhibit initial attachment by altering cell membrane function [23]. Amino acids like D-amino acid, L-tryptophan and glutamate trigger biofilm disassembly inducing biofilm dispersion [24]. Indole and its derivatives affect transcription in pathogenic *E. coli* [25]. Metal chelators act by increasing the sensitivity of cells to biocidal agents [26], nitric oxide donors induce biofilm dispersion [27], sometimes a combination of two or more techniques also used for biofilm inhibition [28].

Material surfaces play a vital role in microbial attachment and propagation, still they are quite often neglected, and only a few guidelines and microbiological assessment protocols are available [29]. To overcome the problem of surface contamination, new strategies are being developed and used for surface modification and functionalization [30]. Use of surfaces with incorporated antimicrobial properties or modified with active biological nanometals are some of the recently proposed strategies [31,32].

In this work, a comparative study of the available catheter materials has been performed for microbial attachment, and their surface properties have been compared to come out with parameters to select the best material resistant to biofilm formation. A new drug-entrapped nanoparticle-based approach to prevent catheter-associated infections has also been tested. The antibiotic gentamicin sulfate (G-S) has been encapsulated in Eudragit RL100, which swells partially at physiological pH, thus acting as decent material for the prolonged drug release [33]. The *E. coli* bacteria are studied for their initial adhesion and ability to form mature biofilm colonies on different catheter biomaterials, namely latex rubber (rubber catheter), silicone rubber (Foley catheter) and polyurethane (endotracheal tube and enteral feeding catheter). Stainless steel (316L) plates, which are commonly used in orthopedic implants, have also been tested for biofilm formation study.

## 2. Results

### 2.1. Biofilm Formation and Quantification

**Figure 1:**
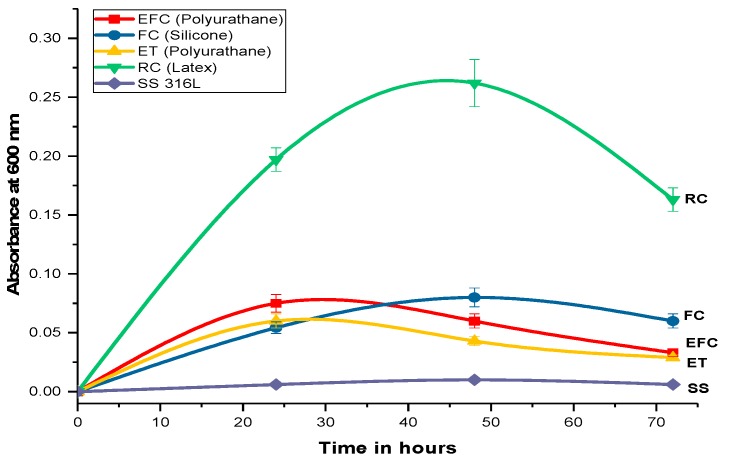
Graphical pattern of *E. coli* biofilm formation over different materials.

Variation of biofilm formation with time is shown in Figure 1. It is seen that the maximum biofilm formation occurred over rubber (latex) and Foley urethral catheter after 48 h of incubation while over enteral feeding and endotracheal catheter after 24 h. Crystal violet assay leads to purple staining of the bacterial cells that are attached to the surface whereas abiotic surfaces are not stained. The rubber (latex) catheter has been found to be a more susceptible surface for biofilm formation, and the bacteria show a greater tendency to form the biofilm. The different patterns of biofilm formation over the EID surfaces indicate that the bacterial biofilm formation depends on the surface properties like texture, grooves, surface charge, and other surface properties of the EIDs.

### 2.2. Physical Characteristic of Indwelling Devices

**Figure 2:**
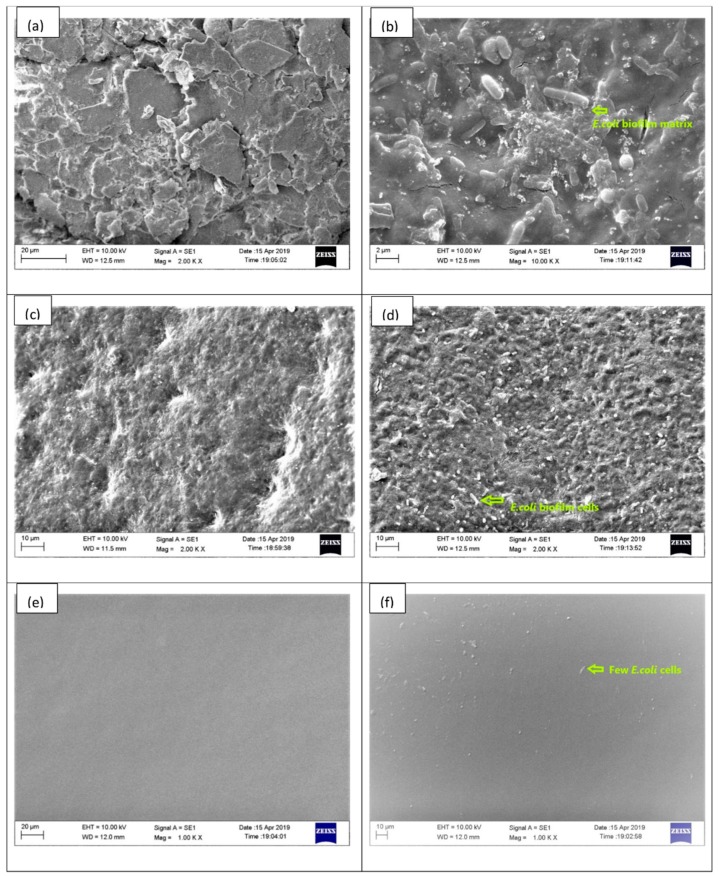
SEM images of catheter inner surfaces before and after *E. coli* biofilm formation; (**a**) rubber catheter; (**b**) biofilm over rubber catheter; (**c**) Foley catheter; (**d**) biofilm over Foley catheter; (**e**) endotracheal catheter; (**f**) biofilm over endotracheal catheter.

The SEM of EIDs surfaces showed differences in polymer surface structures (Figure 2). The external surface of rubber (latex) is more irregular, with grooves (Figure 2a), thus it provided a better site for microbial attachment and caused rich biofilm formation over it (Figure 2b). Dense rod-shaped *E. coli* bacterial colonies can be visualized on the rubber catheter surface. The Foley catheter made of silicone also has surface irregularities (Figure 2c), but the depressions are not as prominent as in case of rubber catheter. The microbial attachment over the surface is clearly visible (Figure 2d). The enteral feeding and endotracheal catheter (polyurethane) surface showed the fewest irregularities (Figure 2e,f) and had few sites available for microbial attachments.

### 2.3. Contact Angle Measurement

**Figure 3:**
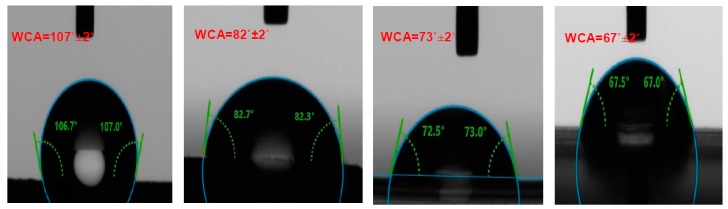
Contact angle of sessile water drop over different catheter surfaces.

The hydrophilic and hydrophobic properties of the surfaces of the EIDs were analyzed with the help of water drop contact angles. The surface with a water contact angle (WCA) <90° has been considered hydrophilic while the surface with WCA >90° considered hydrophobic. The latex rubber catheter surface is hydrophobic while the Foley catheter (silicone rubber) and the endotracheal tube and enteral feeding catheter made of polyurethane surfaces are hydrophilic (Figure 3). The stainless steel (316L SS) plate used for the biomedical application also showed hydrophilic nature. Considering the nature of the surfaces and WCA, the order of hydrophilic nature is stainless steel > polyurethane > silicone rubber > latex rubber and the same is the order for *E. coli* biofilm formation over the specimens suggesting the possible interdependence between the nature of the material and biofilm formation apart from the other physical factors.

### 2.4. Physical Characteristic of Nanoparticles

**Figure 4:**
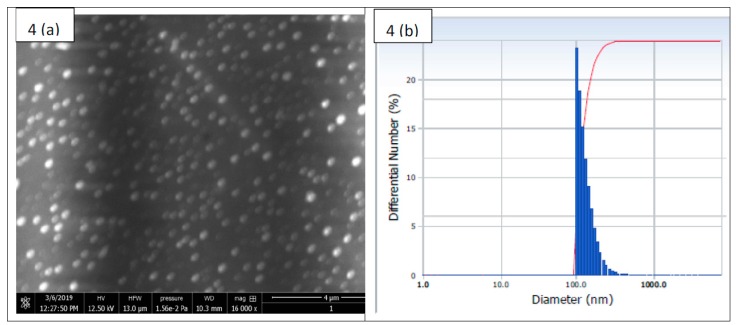
Size distribution of Eudragit RL-100 encapsulated gentamicin sulfate (E-G-S) nanoparticles. (**a**) SEM image; (**b**) Dynamic light scattering (DLS) measurement of particle size distribution.

The SEM image of nanoparticle-encapsulating drugs showed that G-S nanoparticles size was around 100–250 nm (Figure 4a). The nanoparticles were uniformly distributed. The particle size was analyzed through dynamic light scattering (DLS) technique and was found in the range of 100–350 nm (Figure 4b).

### 2.5. Drug Entrapment Efficiency

Initially, the study started with G-S concentration of 142.8571 μg/mL, out of which only 100.2256 μg/mL got entrapped in Eudragit RL100 nanoparticles and 42.6315 μg/mL remained unentrapped resulting in drug entrapment efficiency of 70.15%. The drug concentration calculations were based on standard equation Y = 0.065X + 0.0129 at 260 nm.

**Figure 5:**
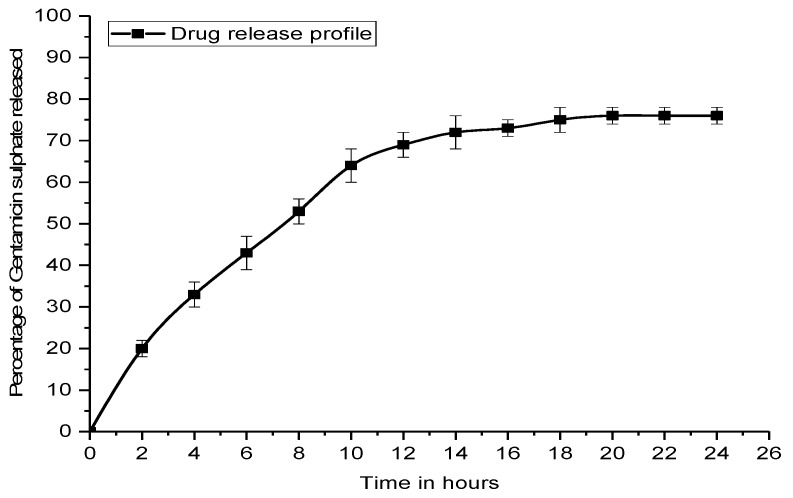
Drug release profile of E-G-S in phosphate-buffered saline (PBS).

In vitro drug release from the nanosuspension in phosphate buffer (pH 7.4) was investigated by the membrane diffusion technique. The sample was taken at regular intervals of 2 h for 24 h. The in vitro drug release profile was obtained from the dialysis experiment. Initially a quick release is seen, probably due to diffusion, but later on, the release slows down and becomes nearly constant after 16 h. More than 70% of the entrapped drug gets released during this period (Figure 5).

### 2.6. Minimum Inhibitory Concentration (MIC) Determination of G-S

**Figure 6:**
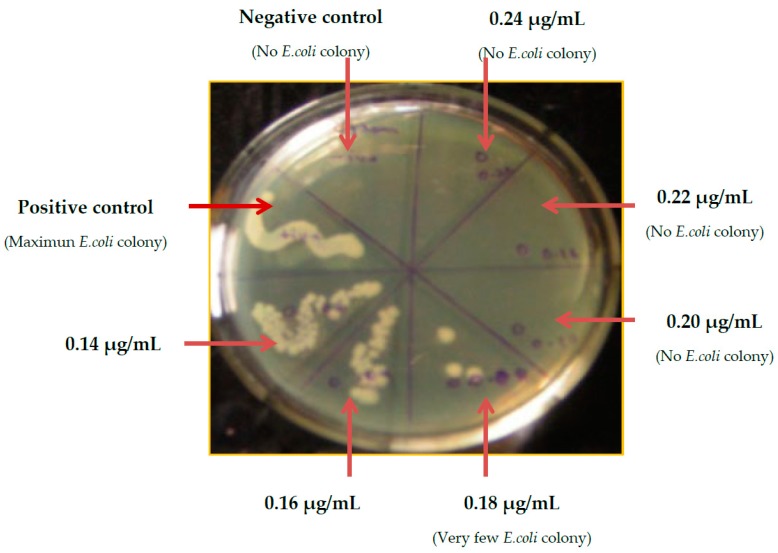
Minimum inhibitory concentration (MIC) observation of G-S for *E. coli* by visual detection of colony forming units (CFU).

The MIC value of G-S for *E. coli* was tested over nutrient agar plates, and the lawns of different overnight grown test culture of *E. coli* with a variable concentration of G-S after incubation at 37 °C for 24 h are shown in Figure 6. On examination of the plate colonies, the growth of *E. coli* was observed up to the concentration of 0.18 µg/mL, but the colony count decreased corresponding to increase of G-S concentration, and no growth was observed at 0.20 and 0.22 µg/mL concentrations. It can be seen that at a G-S concentration above 0.20 µg/mL, the *E. coli* bacteria were unable to grow, thus confirming that the MIC value of G-S for *E. coli* bacteria is 0.20 µg/mL. Agar plate method provides qualitative confirmatory test MIC of G-S.

### 2.7. Minimum Biofilm Inhibitory Concentration (MBIC)

**Figure 7:**
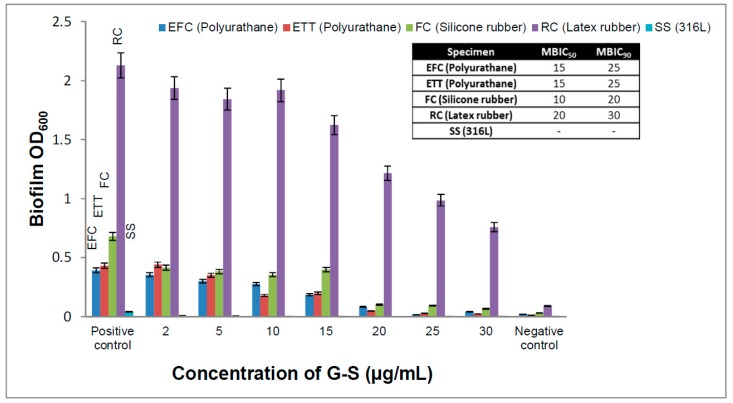
Biofilm inhibition pattern over different externally implanted medical devices (EIDs).

Biofilms are structured communities that are unable to show any explanatory drug resistance by any single mechanism, and complete reduction or inhibition is difficult to achieve [20]. MBIC is the lowest concentration of drug that can reduce the microbial biofilm by 50% (MBIC_50_) or 90% (MBIC_90_). Based on percentage reduction in OD_600_ of spectrophotometric data MBIC_50_ for G-S in the case of the rubber, Foley, and enteral feeding catheters were found to be 20 µg/mL (100 times that of MIC), 10 µg/mL (50 times that of MIC), and 15 µg/mL (75 times that of MIC), respectively, while MBIC_90_ for G-S in the case of the rubber, Foley, and enteral feeding catheters was found to be 30 µg/mL (150 times that of MIC), 20 µg/mL (100 times that of MIC), and 25 µg/mL (125 times that of MIC), respectively (Figure 7). It is seen that the MBIC value for EIDs was much higher as compared to MIC value under similar conditions for the planktonic *E. coli*. The results demonstrate that in case of sessile microbial biofilm, the drug added was not fully available to the microbes, and a major portion of drug/antimicrobial agent was generally reacted and inactivated by chemicals or enzymes of EPS matrix [11]. Polymeric matrix forms a physical barrier and prevents drug penetration, thus protecting sessile microorganisms from being killed [28]. The adaptation acquired by bacterial cells in the biofilm makes it difficult for the antibiotic to act over the biofilm bacterial colonies.

### 2.8. Determination of MBIC of Eudragit RL100 Nanoparticle-Encapsulated G-S

**Figure 8:**
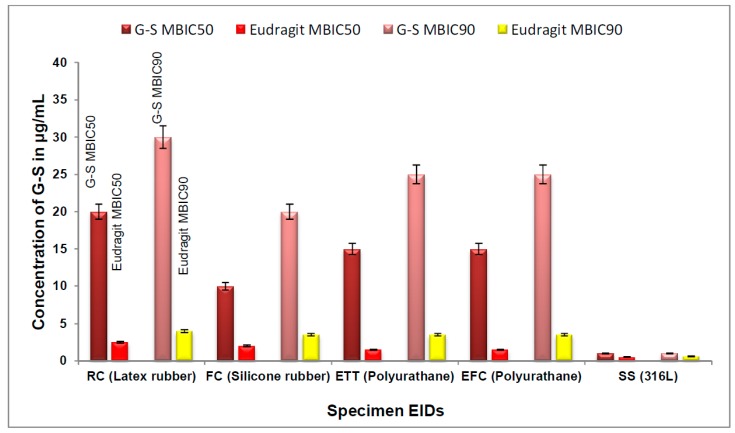
Comparision of minimum biofilm inhibitory concentration (MBIC): MBIC_50_ and MBIC_90_ of G-S and E-G-S.

The MBIC_50_ and MBIC_90_ values were calculated for E-G-S, which was used against biofilm in this study. The values for MBIC_50_ for the nanoparticle-mediated drug delivery for latex, silicone, and polyurethane catheters were found to be 2.5, 2.0, and 1.5 µg/mL, respectively. These concentrations are very low when compared to those of normal unentrapped antibiotics (Figure 7). Similarly, the values for MBIC_90_ for the nanoparticle-mediated drug delivery over latex, silicone, and polyurethane were found to be 4.0, 3.5, and 3.5 µg/mL, respectively (Figure 8). These concentrations are very much lower than those for unentrapped antibiotics. Nanoparticle-encapsulating G-S MBIC_50_ and MBIC_90_ were about 8 and 25 times lower than respective MBIC for unentrapped G-S for latex (rubber catheter), 5 and 6 times lower for silicone (Foley catheter) and 15 and 7 times lower for polyurethane (enteral feeding and endotracheal catheter).

## 3. Discussion

The formation of biofilm is not uniform among the EIDs. Factors such as hydrophobicity, the surface charge of microorganisms, and material from which catheter is made, size, shape, water contact angle, and topographical features of the surface also play a role in microbial adherence to the catheter surface [34]. The maximum tendency for biofilm formation in the current study occurs over the latex rubber catheter followed by silicone rubber Foley catheter. In both cases, the duration for maximum biofilm formation is 48 h. This may be due to their surface roughness, cracks, and fissure, which is visible in SEM images (Figure 2). The depressions and irregularities allow the *E. coli* bacteria to grow their colony up to a greater extent as they provide a site for microbial adhesion, and the complex interplay between biofilm matrix and the catheter surface delays the detachment phase of biofilm. On the positive side, it may be said that although these surfaces are more prone to biofilm formation, they delay the bacterial migration preventing its further spread for a longer duration, and the ill effect may confine to a local area. In contrast with other catheters, enteral feeding and endotracheal catheters (polyurethane) showed comparatively smoother surfaces with fewer cracks and grooves. Only a few colony forming units (CFU) are seen in SEM images (Figure 2f), and the maximum biofilm occurs after 24 h. It is in accordance with our assumptions that smooth surfaces do not provide an easy bacterial attachment, and if the bacteria get attached, it detaches easily to spread to other sites. Stainless steel 316L is very smooth, and it hardly provides any bacterial attachment sites. This is why negligible biofilm formation occurred.

The water contact angle analysis reveals that increase in hydrophilicity of EID surfaces has a direct relation with the biofilm formation, i.e., moderate hydrophobic biomaterial surfaces provide a better site for microbial proliferation and the hydrophilic biomaterial surfaces are less prone to biofilm formation. This finding is in accordance with Yue et al. [35], in that a moderate hydrophobicity with water contact angle around 90° is suitable for bacterial adhesion. Recently superhydrophobic surfaces are reported for reduced bacterial adhesion due to the maintenance of entrapped air between the liquid and solid surface by reducing interface contact area and adhesion forces. This reduction in adhesion forces results in easy detachment of bacterial cells. However, if the entrapped air are intruded by the bacterial media, then the roughness and increased effective attachment area result in adherence of more bacterial cells leading to prominent bacterial colonies [35,36]. The negatively charged bacterial surface also plays a role for the bacterial attachment; the more positive the charge of the surface, the greater the tendency of the microbe for surface attachment [34].

The size of E-G-S as measured by DLS technique (Figure 4b) [37] shows that the average hydrodynamic particle diameter is 133.9 ± 40.9 nm in number distribution. The cumulative % distribution of E-G-S from the DLS study reveals that a cumulative 20% of the particles are below the size of 99.9 nm, 40% are below 109.6 nm, 60% are below 123.6 nm, 80% are below 148.3 nm, 95% are below 202.6 nm.

The G-S entrapment efficiency of Eudragit RL-100 is 70.15%, which is very good because there is extended-release time up to 16 h, and about 70% of the G-S gets released by that time. Thus, if we see the larger picture, nearly 50% of the initial amount of drug is going to be utilised for the biofilm eradication and prevention. If we compare and analyze the values, MBIC_50_ and MBIC_90_ of unentrapped G-S and E-G-S (Figure 7 and Figure 8), the latter is 5–10 times more efficient in removing the biofilm. A smaller dose of E-G-S will be able to prevent and eradicate biofilm effectively.

Thus, the E-G-S is efficient in preventing and eradicating the biofilm over EIDs and is also safe as it reduces the biological payload of antibiotics. It also helps to reduce the tolerant bacterial infection within the subject’s body. Complete removal of formed biofilm is difficult to achieve, but the Eudragit RL-100 nanoparticle-mediated drug delivery can efficiently act against the *E. coli* biofilms. Lower doses of the drug are needed as the nanoparticles can easily penetrate the EPS to act directly on the target site without exceeding the systemic toxicity value of the drug and preventing possible side effects [38]. The polymeric biomaterial surfaces are differentially susceptible to microbial colonization based on their surface properties, and E-G-S nanoparticles offer a better perspective for catheter-associated biofilm removal.

## 4. Materials and Methods

### 4.1. Materials and Reagents

Eudragit RL-100 was purchased from Evonik Rohm GmbH Darmstadt, and gentamicin sulfate (G-S) and mannitol were purchased from Sigma-Aldrich, Saint Louis, MO, USA. Polyvinyl alcohol (PVA), methylene dichloride (MDC), nutrient agar, phosphate-buffered saline (PBS), nutrient broth, lysogeny broth (LB), crystal violet (CV), dimethyl sulfoxide, ethanol, disodium hydrogen phosphate, potassium dihydrogen phosphate were purchased from Himedia Laboratories Pvt. Ltd., Mumbai, India. The standard strain of *E. coli* ATCC 25922 was procured from Institute of Medical Sciences, Banaras Hindu University, Varanasi, India, was used as the reference in this study. Externally implanted medical devices (EIDs)/catheters (enteral feeding catheter (Ramsons Scientific & Surgical Industries Pvt. Ltd., Agra, India), rubber catheter, Foley catheter (Ramsons Scientific & Surgical Industries Pvt. Ltd., Agra, India) and stainless steel plates-(316L) (Zealmax Innovations Pvt. Ltd., Ahmedabad, India)) were obtained from local medical shops. All solvents and chemicals used were of analytical grade. The borosilicate glass-wares used in experiments were acid-washed and rinsed with double-distilled (DD) water followed by heat sterilization before use.

### 4.2. Biofilm Formation and Quantification

Biofilms were formed on the EIDs using the microtiter dish biofilm formation assay [39]. *E. coli* strain was cultured in LB at 37 °C for 24 h, and the cell density was set at 1.5 × 10^8^ cell/mL using hemocytometer. One mL of 1.5× 10^8^ CFU/mL of *E. coli* suspension was added to a 2 mL capped eppendorf tube having (0.5 × 0.5) cm^2^ EIDs and incubated at 37 °C for 90–120 min (adhesion phase). After incubation, EIDs were washed with PBS buffer, and then a fresh broth was added to the catheters, and subsequently incubated at 37 °C for 24, 48, and 72 h for biofilm formation. Timely quantification of biofilm was done by crystal violet assay [40]. The 0.5% (*w*/*v*) CV solution in DD water was applied to the catheter pieces, and subsequently incubated for 30 min at room temperature. After 30 min of incubation, the blocks were rinsed thrice with PBS to remove any unbound dye. Later the CV was washed out from the bacterial biofilms over the catheter surfaces with 96% ethanol, and the resultant solution was spectrophotometrically analyzed for the absorption of the resulting violet solution at 600 nm using 96% ethanol as the blank [41].

### 4.3. Determination of Minimum Inhibitory Concentration (MIC) of G-S

The MIC of G-S was determined by the broth dilution method [42]. One mL of 1.5 × 10^8^ CFU/mL of *E. coli* in LB was taken in 2 mL eppendorf tubes. Different concentrations of G-S (0.10, 0.12, 0.14, 0.16, 0.18, 0.20, 0.22, 0.24 µg/mL) were then added to the eppendorfs. The tube containing LB and test organism (without G-S) served as the positive control, while the tube containing only LB served as the negative control. The capped tubes were then incubated with constant shaking at 37 °C for 24 h. After incubation, the broth was streaked onto a sterile nutrient agar plate and again incubated at 37 °C for 24 h and the nutrient agar plate was examined for the absence of visual colonies for the minimum G-S concentration. The sample in the eppendorf tubes was tested spectrophotometrically for visible turbidity at 600 nm to verify the authenticity of the MIC test.

### 4.4. Determination of Minimum Biofilm Inhibitory Concentration (MBIC) of G-S

To determine the MBIC value of G-S different concentrations, 2, 5, 10, 15, 20, 25, 30 µg/mL of G-S were used. The EID pieces of (0.5 × 0.5) cm^2^ containing 24 h grown biofilm were washed thrice and incubated at 37 °C for 24 h along with positive control samples (without antibiotics). After incubation, the EID pieces were analyzed for biofilm quantification by the crystal violet assay method described earlier. The spectrophotometric absorbance at 600 nm for biofilm was compared with a positive control to find out the MBIC values. Each MBIC experiment was conducted simultaneously with MIC determination to reduce the error.

### 4.5. Preparation of Eudragit RL100 Nanoparticle-Encapsulating G-S

Nanoparticle-encapsulated G-S was prepared by the solvent displacement or nanoprecipitation technique [33]. The PVA solution of known concentration (5% *w*/*v* in 100 mL distilled water) was prepared and stirred at room temperature for 10 min to obtain a homogeneous solution. One hundred mg of G-S was mixed in this solution. In a fresh Erlenmeyer flask, 5 g of Eudragit RL-100 was dissolved in 100 mL of methylene dichloride (MDC) and sonicated for 5 s. The G-S solution was added to the sonicated Eudragit RL-100 solution and further sonicated for 25 s to form a water in organic emulsion (*w*/*o*). A fresh 250 mL of PVA solution (1% *w*/*v*) was then added in water in an oil emulsion to form multiple emulsion (*w*/*o*/*w*). The resultant emulsion was homogenized at 15,000–20,000 rpm thrice to form the Eudragit RL-100 nanoparticle encapsulated drug. Two hundred and fifty mL of PVA (0.3% *w*/*v*) was prepared in DD water and mixed with multiple emulsion and placed on the magnetic stirrer for 6 h at 25 °C at 200 rpm to evaporate the MDC and organic solvents. To get the nanoparticle in powder form, deep-frozen samples were placed in a lyophilizer at −40 °C until the dried powdered sample was obtained.

### 4.6. Drug Entrapment Efficiency

A 20 mL solution of the prepared nanosuspension was centrifuged at 18,000× *g* for 2.5 h at 10 °C, using Remi C-24 cooling centrifuge. The proportion of unentrapped drug was estimated by taking the absorbance of the appropriately diluted supernatant solution at 260 nm using UV-Visible Spectrophotometer (Systronics, Model 2202, Ahmedabad, India) against blank/control nanosuspension. By subtracting the amount of G-S in supernatant from the initial amount of the G-S taken, entrapment efficiency of nanoparticle was calculated using the formulas

(1) $$\mathrm{Entrapment}\;\mathrm{efficiency}\;=\frac{\mathrm{Amount}\;\mathrm{of}\;\mathrm{drug}\;\mathrm{entrapped}\;\mathrm{in}\;\mathrm{nanoparticle}}{\mathrm{Initial}\;\mathrm{amount}\;\mathrm{of}\;\mathrm{drug}\;\mathrm{used}}\;\times \;100$$

Amount of drug entrapped = Initial amount of drug used – Drug present in the supernatant.

### 4.7. In Vitro Drug Release Kinetic Study

The in vitro drug release kinetics of the formed nanoparticle were studied by the static Franz diffusion cell [43]. A dialysis membrane made of cellulose acetate of 25 mm diameter was placed at the terminal portion of the donor compartment. A 10 mL portion of the nanosuspension with the drug was placed into the donor compartment to act as a source. The receptor compartment was filled with 90 mL of 0.2 M phosphate buffer solution of pH 7.4 maintained at 37 °C under mild agitation using a magnetic stirrer. At predefined specific time intervals, aliquots of 1 mL were withdrawn and immediately maintained with the same volume of fresh phosphate buffer solution. The amount of drug released was estimated by taking the absorbance at 260 nm using a UV spectrophotometer.

### 4.8. Scanning Electron Microscopy (SEM)

The scanning electron microscopic (SEM) analysis was performed for determining the size of the formed nanoparticles, the sample’s surface morphology of EIDs, and bacterial biofilm. All the EID samples [(0.5 × 0.5) cm^2^] were pasted onto the sample holder, and E-G-S nanoparticles were allowed to dry at ambient temperature before the measurement using scanning electron microscopy (FEI, Quanta 200F, Tokyo, Japan) at an acceleration voltage of 10 kV. Lyophilized E-G-S samples were also analyzed for their size using Dynamic Light Scattering method (Particulate Systems, Nanoplus particle size analyzer, Norcross Georgia, GA, USA) [37].

### 4.9. Contact Angle

The hydrophilicity and hydrophobicity of the EID surfaces were evaluated by the surface contact angle measurement between the sessile water drop and EID surface. The contact angle measurement for water was performed by using contact-angle-measuring drop-shape analyzer (DSA25S, KRUSS, Hamburg, Germany). A drop of water (20 μL) was dropped over the piece of catheter material using an automatic microsyringe, and then static images for each surface were taken [44]. In each case, the angle was measured thrice, and the average value was taken as the contact angle value of that material surface.

### 4.10. Antimicrobial Assessment

*E. coli* cells were grown at 37 °C for 24 h in LB media. Cell number was determined by using a hemocytometer and agar plate counting method and adjusted to 1.5 × 10^8^ CFU/mL. Different concentrations of G-S were used to analyze the antimicrobial property.

### 4.11. Statistical Analysis

All data sets are measured and represented as mean standard deviation (SD), and experiments have been performed at least in triplicate.

## 5. Conclusions

Material characteristics and surface properties are directly related to microbial adhesion, colonization and detachment. Irregularities on the polymeric surface promote biofilm formation because of the increased effective surface area for microbial attachment. The rough surfaces and depressions in the uneven surfaces provide shelter and site for microbial colonization. In vitro studies have confirmed that both adherence of biofilm and decrease in hydrophilicity increase in the order: Stainless steel 316L < polyurethane < silicone < latex for Gram-negative *E. coli,* thus justifying the differential susceptibility of polymer surfaces for the microbial colonization. The E-G-S nanopowder has better protective property against the biofilm, so its coating or its powdered form is very effective against prevention of biofilm-related infections. The study also confirms that the hydrophobic surfaces in the moderate range are more prone to bacterial cell attachment and biofilm formation, and the tendency to form biofilm over the biomaterial decreases with increase in hydrophilicity. The polyurethane polymer is found to be most suitable for the EIDs as compared to silicone and latex rubber.

## Abbreviations

EIDs	Externally implanted medical devices
E-G-S	Eudragit RL100 encapsulated gentamicin sulfate
G-S	Gentamicin sulfate
